# Small Wonders: How Nanotechnology Is Redefining Shoulder and Elbow Surgery

**DOI:** 10.3390/cells15191830

**Published:** 2026-10-08

**Authors:** Alexander Shao-Rong Pang, Joel Louis Lim, Dinesh Kumar Srinivasan, Zi Qiang Glen Liau

**Affiliations:** 1 Yong Loo Lin School of Medicine, National University of Singapore, Singapore 117597, Singapore; alexandersrp@u.nus.edu; 2 Department of Orthopaedic Surgery, National University Hospital, Singapore 119074, Singapore; joel_louis_lim@nuhs.edu.sg (J.L.L.); glen_liau@nuhs.edu.sg (Z.Q.G.L.); 3 Department of Anatomy, Yong Loo Lin School of Medicine, National University of Singapore, Singapore 117594, Singapore

**Keywords:** nanotechnology in orthopedics, rotator cuff repair augmentation, bioinductive scaffolds, osseointegration, biomimetic nanomaterials

## Abstract

Objective: Nanotechnology has been increasingly incorporated into shoulder and elbow surgery, from bioinductive collagen scaffolds for rotator cuff repair to nanostructured implant surfaces for arthroplasty and nanoparticle-functionalized sutures for elbow tendon repair. This review evaluates the current evidence base for these applications and clarifies which technologies are supported by clinical data versus preclinical/mechanistic evidence alone. Methods: A narrative review was conducted using PubMed/MEDLINE, Embase, Cochrane Library, Web of Science, and Scopus (January 2005–September 2026), supplemented by manual screening of society guidelines. Of 1842 records identified, 77 articles and 6 guideline documents were considered in the initial evidence synthesis, with 54 references ultimately retained in the published review. Because of heterogeneity in study design (randomized trials, cohorts, and preclinical models), findings were synthesized narratively. Results: In rotator cuff repair, bioinductive collagen (REGENETEN) and acellular dermal matrix augmentation have the most mature clinical evidence, with randomized and cohort data showing reduced retear rates, whereas the collagen-PLLA biocomposite (BioBrace) remains largely preclinical. In shoulder and elbow arthroplasty, nanostructured and nanotextured titanium surfaces have preclinical and early clinical support for enhanced osseointegration, while antimicrobial silver and titanium dioxide nanocoatings and metal-phenolic network coatings remain supported chiefly by in vitro and animal data, with cytotoxicity and photocatalytic limitations still unresolved. In elbow surgery specifically, nanoparticle- and gene-functionalized sutures have shown benefit in flexor tendon models with anatomically agnostic translational potential, but direct elbow-specific clinical data are lacking, and applications to the ulnar collateral ligament, cartilage, and total elbow arthroplasty bearings remain at the preclinical or expert-opinion stage. Conclusions: Nanotechnology is clinically established only for a small number of shoulder and elbow applications, principally bioinductive collagen augmentation of rotator cuff repair; most other applications, including antimicrobial nanocoatings and elbow-specific nanoparticle delivery systems, remain mechanistically promising but clinically unproven. Progress toward clinical translation will depend on adequately powered trials that separate device-specific effects from the heterogeneous preclinical literature currently supporting the field.

## 1. Introduction

Nanotechnology, defined as the study and manipulation of materials at the nanoscale (typically 1–100 nm), has emerged as a transformative force in orthopedic surgery over the past two decades. At this dimensional scale, materials exhibit unique quantum mechanical properties that fundamentally alter their behavior in terms of reactivity, conductivity, and biological interactions [1]. The dramatic increase in surface area-to-volume ratio at the nanoscale enables enhanced cellular interactions, making nanotechnology particularly valuable for improving implant-tissue interfaces and promoting tissue regeneration [1,2,3].

In shoulder and elbow surgery, nanotechnology has revolutionized multiple aspects of care, from enhancing rotator cuff repair outcomes to improving osseointegration in arthroplasty procedures. The field has evolved from basic surface modifications to sophisticated biomimetic scaffolds that actively promote tissue regeneration while preventing complications such as infection and implant failure [4].

**Figure 1:**
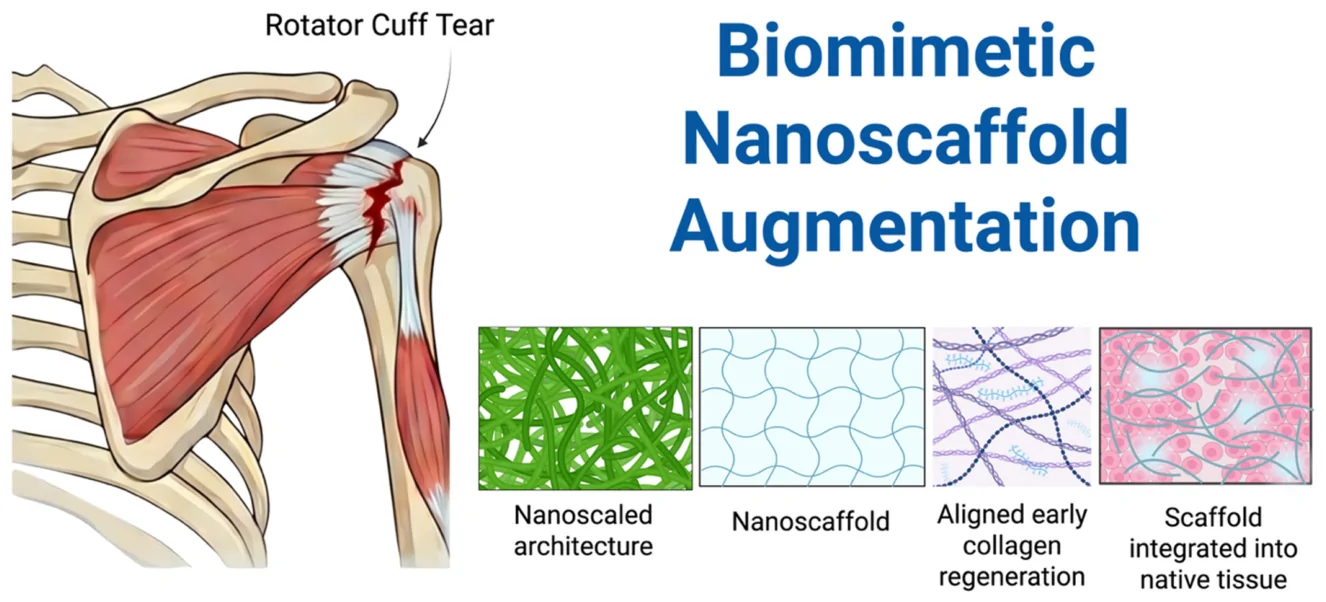
The biomimetic rationale for nanotechnology in tendon repair. Created in BioRender. Pang, A.S.-R. (2026) https://BioRender.com/956gllk (accessed on 27 September 2026).

The biological rationale for nanotechnology in upper extremity surgery is compelling. Native extracellular matrix (ECM) components, including collagen fibrils and proteoglycans, exist at the nanoscale, typically ranging from 10 to 300 nm in diameter [5,6]. By engineering materials that mimic these natural structures, surgeons can create scaffolds that more closely replicate the native tissue architecture (Figure 1), thereby facilitating improved cellular recognition, attachment, and differentiation [7,8]. This biomimetic approach has proven particularly valuable in rotator cuff repair, where the challenge of achieving durable tendon-to-bone healing has historically resulted in retear rates as high as 94% in some patient populations [5].

Strictly defined, nanotechnology refers to structures or engineered features with at least one dimension in the 1–100 nm range, such as collagen fibrils, electrospun nanofibers, nanoparticles, and nanotextured implant surfaces. This review restricts the term “nanotechnology” or “nanostructured” to materials meeting that definition, and specifies the relevant nanoscale feature (fibril diameter, particle size, surface texture) wherever such a material is discussed. Several widely used clinical products discussed here, including acellular dermal matrix (ADM), REGENETEN, and the collagen component of BioBrace^®^, are bulk decellularized or manufactured collagen scaffolds; these are included because their functional collagen fibrils and pores fall partly within or immediately adjacent to the nanoscale (fibril diameters approximately 50–200 nm), and because they are the clinical products through which nanoscale collagen engineering has been translated into shoulder and elbow surgery. Micron-scale features that exceed 100 nm, such as the 20–200 μm bulk pore architecture of REGENETEN or micro-textured implant surfaces, are discussed for procedural and structural context but are explicitly identified as microscale rather than nanoscale and are not themselves classified as nanotechnology in this review. Where a product or technique combines both scales (e.g., a micron-scale pore network built from nanoscale collagen fibrils), the nanoscale and microscale components are distinguished explicitly rather than treated interchangeably.

This review examines the current state of nanotechnology applications in shoulder and elbow surgery, with particular emphasis on rotator cuff repair augmentation techniques, arthroplasty innovations, and emerging applications in elbow pathology.

## 2. Methodology

This is a narrative review, and the methodology below is provided to give readers transparency regarding the scope of evidence considered rather than to satisfy the reporting standards expected of a systematic review or meta-analysis.

Databases including PubMed/MEDLINE, Embase, Cochrane Library, Web of Science, and Scopus were searched, supplemented by manual screening of reference lists of included articles and of relevant society guideline documents (American Academy of Orthopaedic Surgeons, American Shoulder and Elbow Surgeons). Articles published between January 2005, and September 2026 were eligible, reflecting the period over which nanoscale surface engineering and bioinductive scaffolds entered orthopedic clinical practice. No lower date limit was applied to seminal preclinical nanomaterial science papers referenced for mechanistic background.

Searches combined controlled vocabulary (MeSH) and free-text terms using Boolean operators, structured around three concept blocks: (1) anatomic site/procedure terms (“rotator cuff repair,” “shoulder arthroplasty,” “reverse shoulder arthroplasty,” “elbow arthroplasty,” “epicondylitis,” “tendon repair”); (2) nanotechnology/biomaterial terms (“nanotechnology,” “nanoparticle,” “nanocoating,” “nanostructured surface,” “nanofiber,” “bioinductive collagen,” “acellular dermal matrix,” “biocomposite scaffold,” “silver nanoparticle,” “titanium dioxide nanocoating,” “metal-phenolic network”); and (3) named products/techniques (“REGENETEN,” “BioBrace,” “human dermal allograft”). Blocks were combined with AND; terms within blocks were combined with OR.

The initial search returned 1842 records across all databases. After removal of 511 duplicates, 1331 titles/abstracts were screened. Of these, 1096 were excluded for lack of relevance to shoulder/elbow surgery, absence of a nanoscale component, or non-eligible publication type, leaving 235 articles for full-text review. Following full-text assessment, 158 articles were excluded (79 for insufficient methodological detail, 44 for duplicate/overlapping cohorts, 35 for indirect relevance), resulting in 77 articles that directly informed the review, supplemented by 6 society guideline documents. Following further refinement and consolidation during manuscript preparation, 54 references were retained and cited in the final published review.

Because of substantial heterogeneity in study design, patient population, and outcome reporting across the included literature, findings were synthesized narratively rather than pooled quantitatively, with emphasis placed on randomized controlled trials (RCTs) and systematic reviews where available, and preclinical data presented explicitly as mechanistic or hypothesis-generating rather than as clinical evidence.

## 3. Rotator Cuff Repair: Nanotechnology-Enhanced Augmentation Strategies

**Figure 2:**
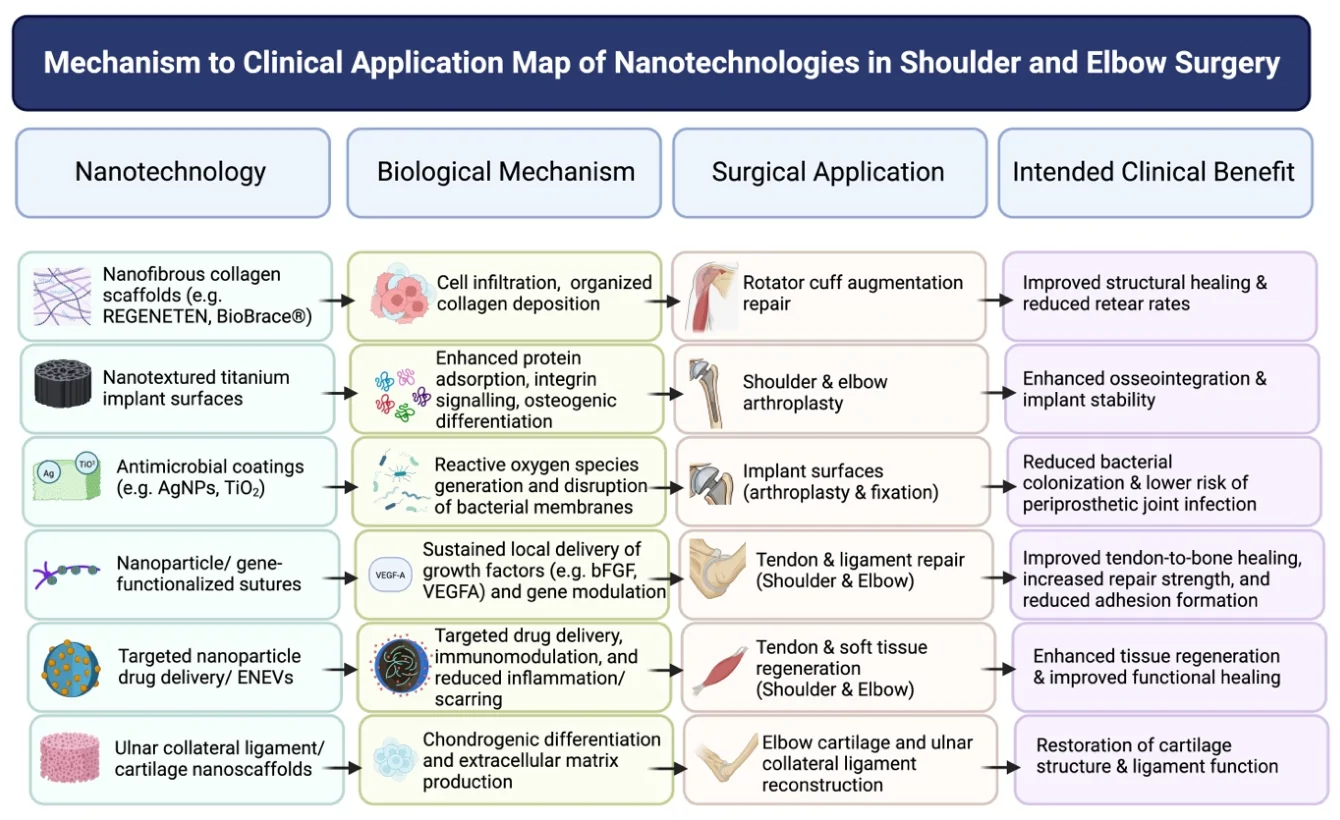
Mechanism to clinical application map of nanotechnologies in shoulder and elbow surgery. Schematic illustration linking specific nanomaterial platforms to their biological mechanisms of action, operative applications, and targeted clinical outcomes across soft tissue repair and arthroplasty. AgNPs, silver nanoparticles; bFGF, basic fibroblast growth factor; ENEVs, engineered extracellular vesicles; TiO_2_, titanium dioxide; VEGF-A, vascular endothelial growth factor A. Created in BioRender. Pang, A.S.-R. (2026) https://BioRender.com/s8x3cq8 (accessed on 27 September 2026).

Taken together, the nanotechnologies reviewed act through a limited set of recurring biological mechanisms across otherwise distinct clinical applications. Figure 2 maps these platforms to their mechanisms of action, operative applications, and targeted outcomes. Not all linkages carry equal clinical validation.

### 3.1. Standard Arthroscopic Repair Without Augmentation

Traditional arthroscopic rotator cuff repair without augmentation serves as the baseline against which nanotechnology-enhanced techniques are measured. Despite advances in surgical technique and anchor technology, retear rates for medium to large tears remain problematic, ranging from 25 to 35% in contemporary series [9,10]. The primary limitation of non-augmented repair lies in the biological healing environment at the tendon-bone interface, where poor vascularity, limited cellular activity, and mechanical stress combine to impede robust tissue regeneration [6,11].

### 3.2. Acellular Dermal Matrix Augmentation

ADM represents one of the earliest biological augmentation strategies, with particular adoption in Korean and other international centers. Human dermal allograft (HDA) patches provide a structural scaffold composed primarily of type I collagen, serving both mechanical reinforcement and biological functions [12,13].

Recent evidence demonstrates significant benefits of ADM augmentation. In a retrospective comparative study of 167 patients with large to massive posterosuperior rotator cuff tears conducted by Kantanavar et al., HDA augmentation reduced retear rates from 29.1% to 5.6% (*p* = 0.007) compared to non-augmented repair. Patients in the augmented group demonstrated superior functional outcomes, with University of California Los Angeles (UCLA) shoulder scores improving to 30.1 ± 4.2 versus 23.2 ± 3.9 in controls (*p* = 0.046), and significantly better forward flexion (138.9 ± 23.5° vs. 127.0 ± 19.5°, *p* = 0.004) [13].

A meta-analysis of RCTs by Hurley et al. examining acellular collagen matrix patch (ACMP) augmentation, which includes ADM products, demonstrated an 11% retear rate with augmentation compared to 34% in controls (*p* = 0.0006) [14]. The American Academy of Orthopaedic Surgeons (AAOS) 2025 guidelines on rotator cuff management cite moderate-quality evidence supporting HDA augmentation, noting improved Constant-Murley scores and reduced retear rates without adverse events [15].

The mechanism of action for ADM extends beyond simple mechanical reinforcement. These scaffolds provide a bioconductive matrix that facilitates cellular infiltration, neovascularization, and gradual remodeling into host tissue [11]. The nanoscale architecture of the collagen fibers within ADM (typically 50–200 nm in diameter) provides topographical cues that guide cellular behavior and promote organized tissue regeneration [6].

### 3.3. REGENETEN Bioinductive Collagen Scaffold

The REGENETEN bioinductive implant (Smith & Nephew, London, UK) represents a significant advancement in nanotechnology-based rotator cuff augmentation. This device consists of a highly porous bovine type I collagen scaffold with a nanofibrous architecture designed to induce new tissue formation through bioinduction rather than simple bioconduction [16,17].

The nanoscale structure of REGENETEN is critical to its mechanism of action. The scaffold features interconnected pores ranging from 20 to 200 μm with collagen fibrils at the nanoscale (approximately 50–100 nm diameter), creating a three-dimensional architecture that mimics native tendon ECM [6,17]. This biomimetic structure facilitates rapid cellular infiltration, promotes angiogenesis, and induces organized collagen deposition aligned with the direction of mechanical stress [16,18].

Clinical evidence for REGENETEN has been particularly robust. A prospective multicenter study of 115 patients with medium to large full-thickness rotator cuff tears conducted by Bushnell et al. demonstrated radiographic retear rates of 11.5% for medium tears and 35% for large tears at 2-year follow-up, with the majority of retears occurring before 3 months. When combined with double-row repair technique, the overall retear rate was only 13.2%. Importantly, over 90% of patients achieved the minimal clinically important difference (MCID) for both American Shoulder and Elbow Surgeons (ASES) and Constant-Murley scores [18].

Two landmark RCTs have established the efficacy of REGENETEN. Ruiz Ibán et al. randomized 124 patients with medium to large posterosuperior rotator cuff tears to transosseous-equivalent repair with or without bioinductive collagen implant (BCI) augmentation. At 12-month follow-up, the BCI group demonstrated an 8.3% retear rate compared to 25.8% in the control group (*p* = 0.010), representing a relative risk reduction of 68% [10]. The 2-year follow-up data confirmed durability of these results, with retear rates of 12.3% in the BCI group versus 35.1% in the control group (*p* = 0.004). Importantly, patients with intact repairs demonstrated superior clinical outcomes compared to those with failed repairs, with better Constant-Murley scores (*p* = 0.035), ASES scores (*p* = 0.015), and pain scores (*p* = 0.006) [9].

A systematic review of 21 studies on BCI augmentation found consistently favorable results, with retear rates ranging from 0 to 9% for full-thickness tears and 0–18% for partial-thickness tears. Tendon thickness increased by 13–44% in full-thickness tears and 14–60% in partial-thickness tears [16]. Two studies demonstrated cost-effectiveness, with savings of $5338–$13,061 per healed rotator cuff tendon due to reduced revision surgery rates. However, a higher rate of adhesive capsulitis was noted with BCI use, suggesting the need for careful patient selection and postoperative rehabilitation protocols [16]. The AAOS 2025 guidelines cite high-quality evidence supporting bioinductive collagen-based tendon implants, noting significantly lower retear rates with augmentation, though functional outcomes did not demonstrate consistent differences in all studies [19].

### 3.4. BioBrace^®^ Biocomposite Collagen-PLLA Scaffold

BioBrace^®^ represents an innovative hybrid approach combining biological and synthetic materials at the nanoscale. This biocomposite scaffold integrates type I collagen with poly-L-lactic acid (PLLA) to provide both bioinductive properties and mechanical reinforcement [20,21].

The nanoscale architecture of BioBrace^®^ is particularly sophisticated. The scaffold features collagen fibers (50–100 nm diameter) interwoven with PLLA nanofibers (200–500 nm diameter), creating a hierarchical structure that mimics the organization of native tendon tissue [20]. This dual-component design leverages the biological activity of collagen for cellular recognition and attachment while utilizing the superior mechanical properties of PLLA to provide structural support during the critical early healing phase [20,21].

Preclinical evidence from an ovine infraspinatus model demonstrated impressive results with a collagen-PLLA biocomposite scaffold. The study evaluated 25 sheep undergoing rotator cuff repair augmented with the biocomposite scaffold, with follow-up extending to 52 weeks. Histology revealed progressive integration and incorporation of new fibrous tissue within, above, and below the scaffold at 6 weeks, with continued maturation and remodeling through 52 weeks. Magnetic resonance imaging (MRI) demonstrated a 180% increase in tendon thickness at all time points compared to non-operative controls. Biomechanical testing showed significant increases in ultimate load between 0, 6, and 12 weeks (*p* < 0.01), indicating progressive strengthening of the repair [21].

The mechanism of action for collagen-PLLA scaffolds involves both immediate mechanical support and gradual biological incorporation. The PLLA component provides tensile strength (354.0 ± 36.0 N dry, 267.2 ± 15.9 N wet) sufficient to maintain normal tendon function during early healing. Simultaneously, the collagen component facilitates cell proliferation and migration, with significantly higher cell coverage on collagen yarns compared to PLLA alone after 28 days of culture. Cells on collagen yarns exhibited multi-directional spreading resembling natural ECM attachment, while cells on PLLA showed elongated morphology along the fiber axis [20].

The biodegradation profile of BioBrace^®^ is designed to match the timeline of tissue regeneration. PLLA degrades slowly over 12–24 months through hydrolysis, providing sustained mechanical support while gradually transferring load to the regenerating tissue. The collagen component undergoes more rapid remodeling (6–12 weeks), being replaced by host-derived ECM as healing progresses [20,21].

### 3.5. Comparative Analysis and Clinical Decision-Making

Biological augmentation strategies demonstrate significant benefits across all modalities. A comprehensive systematic review of 62 studies (47 animal models, 15 clinical studies) by Mandalia et al. found that 87.2% of animal studies demonstrated biomechanical and histologic enhancement with improved load to failure, stiffness, and strength. Among clinical studies, 66.7% showed improvement in retear rates, radiographic thickness, and functional scores. Pooled analysis revealed significantly lower odds of retear with biological augmentation compared to non-augmented repair (odds ratio 0.28, *p* < 0.00001, I^2^ = 0.11) [11].

The choice among augmentation strategies depends on multiple factors including tear size, tissue quality, patient age, and activity level. Current evidence suggests that acellular human dermal allograft and bovine collagen scaffolds demonstrate the most promising preliminary evidence in their respective categories [11]. The AAOS guidelines provide moderate-strength recommendations for both HDA and bioinductive collagen implants, with the caveat that functional outcomes may not differ despite improved structural healing [19].

## 4. Nanotechnology in Shoulder Arthroplasty

### 4.1. Nanostructured Surface Modifications for Enhanced Osseointegration

**Figure 3:**
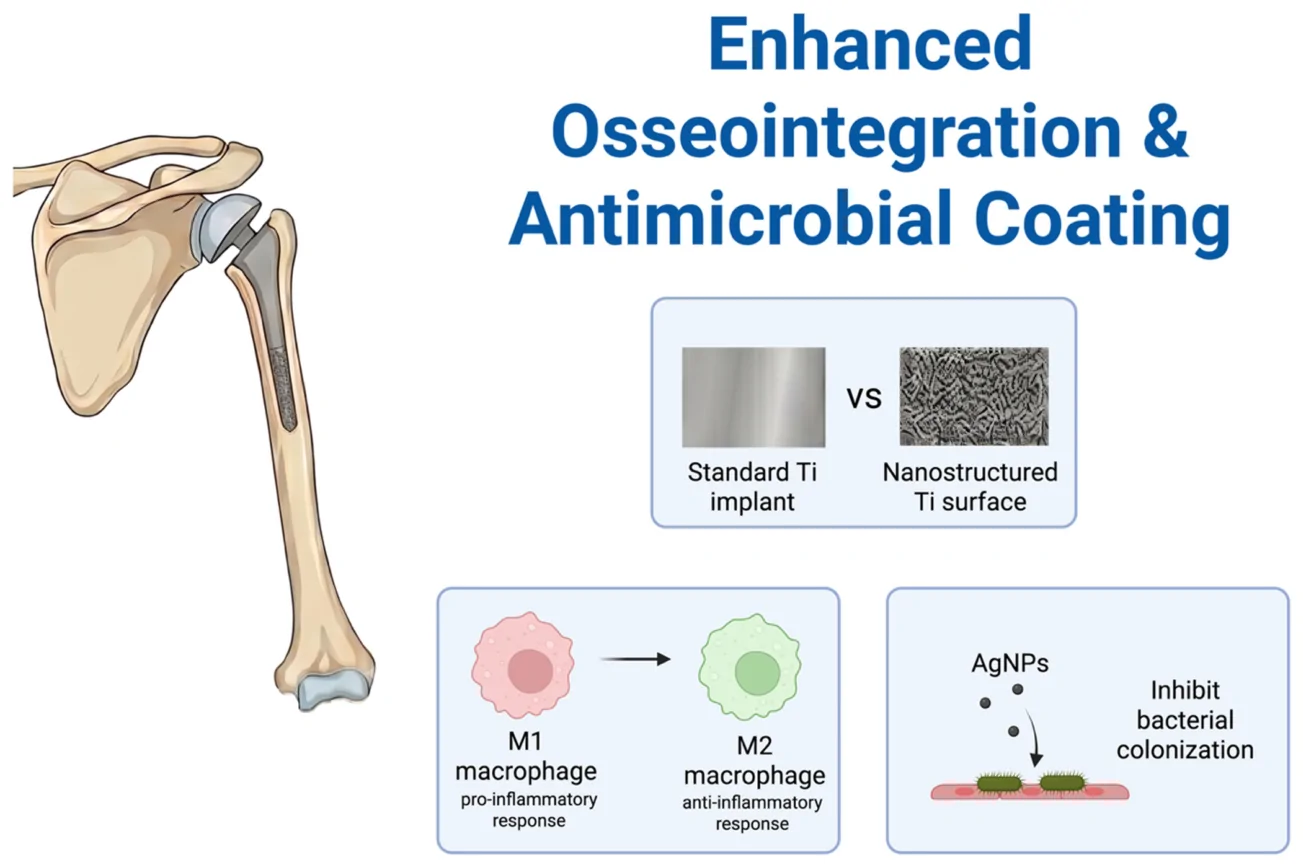
Enhanced osseointegration and antimicrobial coatings in shoulder arthroplasty. Created in BioRender. Pang, A.S.-R. (2026) https://BioRender.com/bnn0djl (accessed on 27 September 2026).

The success of shoulder arthroplasty, whether anatomic total shoulder arthroplasty (aTSA) or hemiarthroplasty, depends critically on achieving rapid and durable osseointegration between the implant and host bone. Nanotechnology has revolutionized this interface through surface modifications that enhance cellular interactions at the molecular level, as illustrated in Figure 3 [1,2,3,7].

Nanostructured titanium surfaces represent a major advancement in implant technology. Modification techniques including anodization, laser texturing, and chemical etching create nanoscale topographies (typically 20–100 nm features) that more closely mimic the architecture of native trabecular bone [1,7]. These nanoscale features dramatically increase the surface area available for protein adsorption and cellular attachment, with the surface area-to-volume ratio increasing as feature size decreases below 100 nm [1].

The biological mechanisms underlying enhanced osseointegration with nanostructured surfaces are multifaceted. Nanoscale topography influences protein adsorption in terms of quantity, species selection, and conformational changes. Specifically, nanostructured TiO_2_ surfaces preferentially adsorb fibronectin and vitronectin in conformations that expose RGD (arginine-glycine-aspartate) sequences, enhancing integrin-mediated cell adhesion [8]. This selective protein adsorption creates a favorable microenvironment for osteogenic differentiation of mesenchymal stem cells (MSCs) [7,22].

A sophisticated micro/nano-biomimetic coating study demonstrated the comprehensive benefits of hierarchical surface structuring. The micro/nanoscale (MNS) titania fiber-like network not only facilitated osteogenic and angiogenic differentiation of bone marrow stromal cells and endothelial cells, but also modulated macrophage polarization, suppressing pro-inflammatory M1 phenotype while stimulating pro-healing M2 phenotype. This immunomodulatory effect created a favorable osteoimmune microenvironment that further enhanced osteo- and angio-genesis through crosstalk of multiple signaling pathways. In vivo evaluation confirmed superior osseointegration with MNS compared to nanoscale-only or pristine titanium implants [22].

A systematic review of nanoscale morphologies on three-dimensional (3D)-printed titanium implants by Yang et al. found that irregular nano-textures, nanodots, and nanotubes with diameters of 40–105 nm significantly improved osseointegration and vertical bone ingrowth compared to untreated surfaces. These nanostructures promoted cell adhesion, matrix mineralization, and osteogenic differentiation through increased integrin expression [23].

### 4.2. Metal-Phenolic Network Nanocoatings

An innovative approach involves metal-phenolic network (MPN) nanocoatings, which combine tannic acid (TA) with bioactive metal ions such as strontium (Sr^2+^). These self-assembled nanocoatings provide a multifunctional bioactive interface that facilitates initial adhesion and recruitment of bone marrow MSCs, polarizes macrophages toward M2 phenotype, and accelerates osteogenic differentiation. In vivo evaluations confirmed enhanced osseointegration through generation of a favorable osteoimmune microenvironment. The facile application process and robust performance make MPN nanocoatings particularly attractive for clinical translation [24].

### 4.3. Antimicrobial Nanocoatings

Periprosthetic joint infection remains a devastating complication in shoulder arthroplasty, with revision rates of 1–3% for infection alone. Nanotechnology offers promising solutions through antimicrobial surface modifications that prevent bacterial colonization without relying on systemic antibiotics [3,25,26].

Silver nanoparticles (AgNPs) represent the most extensively studied antimicrobial nanotechnology in orthopedics [25,27]. AgNPs exert broad-spectrum antimicrobial effects through multiple mechanisms including: (1) disruption of bacterial cell membranes through electrostatic interactions, (2) generation of reactive oxygen species (ROS) that damage bacterial DNA and proteins, (3) interference with bacterial enzyme function, and (4) inhibition of biofilm formation [27]. The nanoscale size (typically 1–100 nm) is critical, as smaller particles demonstrate greater antimicrobial efficacy due to increased surface area and enhanced cellular penetration [25,27].

Clinical applications of AgNPs in shoulder arthroplasty include incorporation into bone cement, hydroxyapatite coatings, and direct surface modification of titanium implants [25]. In vitro studies demonstrate effectiveness against common orthopedic pathogens including *Staphylococcus aureus*, methicillin-resistant *Staphylococcus aureus* (MRSA), and *Escherichia coli* [28]. However, concerns regarding potential cytotoxicity to osteoblasts and fibroblasts necessitate careful optimization of AgNP concentration and release kinetics [27,29].

Titanium dioxide (TiO_2_) nanocoatings offer an alternative antimicrobial strategy with potentially superior biocompatibility. Atomic layer deposition (ALD) of nano-TiO_2_ thin films creates nanostructured surfaces that simultaneously enhance osteoblast adhesion and proliferation while inhibiting bacterial colonization [28]. The antimicrobial mechanism involves photocatalytic generation of ROS under UV light exposure, though this requirement limits applicability for deeply implanted devices. Optimization of surface energy (approximately 38.79 mJ/m^2^) achieved the best balance between antibacterial properties and cellular biocompatibility [1,28].

### 4.4. Clinical Outcomes of Stemless Shoulder Arthroplasty

The application of nanotechnology principles to stemless shoulder arthroplasty design has yielded encouraging long-term results. A study of the Comprehensive Nano reverse total shoulder arthroplasty (rTSA) system (Zimmer Biomet, Warsaw, IN, USA) evaluated 35 shoulders at mean follow-up of 106 ± 14.6 months. Despite a relatively high revision rate (11.4%) primarily due to early mechanical complications, the remaining implants demonstrated excellent clinical outcomes with mean Constant-Murley score of 82.9 ± 13.1, Disabilities of the Arm, Shoulder, and Hand (DASH) score of 10.6 ± 17.3, and subjective shoulder value of 85.0 ± 18.1. Active flexion and abduction averaged 159.8° ± 13.8° and 155.9° ± 20.1°, respectively [30].

Long-term evaluation of the Total Evolutive Shoulder System (TESS) (Zimmer Biomet, Warsaw, IN, USA) and Nano stemless shoulder arthroplasty systems at mean 10-year follow-up (range 8–15 years) demonstrated excellent survivorship and functional outcomes. Statistically significant improvements were observed in range of motion, pain relief, and function as measured by Western Ontario Osteoarthritis of the Shoulder (WOOS) and ASES scores. Radiographic analysis revealed progressive lucency > 2 mm around the glenoid component in 15% of TSA cases, with 11% showing progressive lucency around the humeral component. Overall revision rate was only 4.3% (2 of 47 arthroplasties), confirming the durability of stemless designs [31].

## 5. Reverse Shoulder Arthroplasty: Nanotechnology Applications

Reverse shoulder arthroplasty (RSA) has revolutionized treatment of rotator cuff arthropathy, massive irreparable rotator cuff tears, and complex proximal humerus fractures. The unique biomechanics of RSA, with medialization and distalization of the center of rotation, create distinct challenges for osseointegration and implant stability that nanotechnology helps address [2,32].

The same nanostructured surface modifications discussed for anatomic arthroplasty apply equally to RSA components. The glenosphere and baseplate benefit from nanostructured titanium surfaces that enhance bone ingrowth and reduce micromotion during the critical early healing period [7,33]. The increased surface area and optimized protein adsorption characteristics of nanostructured surfaces are particularly valuable in RSA, where bone quality is often compromised due to advanced age, osteoporosis, or prior surgery [22,24].

Specific challenges in RSA include scapular notching, baseplate loosening, and acromial stress fractures. Nanotechnology-enhanced osseointegration may help mitigate these complications by achieving more rapid and robust fixation [2,4]. The long-term data on the Nano reverse shoulder system, while showing some early mechanical complications, demonstrated that successfully integrated implants achieved excellent functional outcomes and durability [30].

Advanced surface modifications combining micro- and nanoscale features show particular promise for RSA applications. The hierarchical structuring provides immediate mechanical interlock at the microscale while promoting biological integration at the nanoscale [7,22]. This dual-mechanism approach may be especially beneficial in the challenging bone quality often encountered in RSA patients [33].

## 6. Nanotechnology in Elbow Surgery

### 6.1. Nanoparticle- and Gene-Functionalized Sutures for Elbow Tendon and Ligament Repair Lateral, and Medial Epicondylitis

Elbow tendon and ligament injuries, including lateral epicondylitis (tennis elbow), medial epicondylitis (golfer’s elbow), and ulnar collateral ligament tears, represent common pathologies of the elbow, and their limited intrinsic healing capacity has made them a focus of orthobiologic and nanotechnology-enhanced treatments [34,35].

Nanoparticle-coated sutures represent an innovative approach to enhance tendon healing in elbow surgery [36,37]. These sutures feature polydopamine-modified surfaces that uniformly absorb nanoparticle/growth factor complexes, enabling sustained local delivery of therapeutic agents directly to the repair site. In flexor tendon injury models, sutures coated with basic fibroblast growth factor (bFGF) and vascular endothelial growth factor A (VEGFA)-loaded nanoparticles significantly increased ultimate strength of repaired tendons at multiple time points. At 6 weeks, the growth factor-releasing sutures demonstrated significantly lower adhesion scores, longer tendon gliding excursion, and decreased work of digital flexion compared to control sutures [36].

Gene therapy delivered via nanoparticle-coated sutures offers even greater potential for elbow tendon repair. Sutures carrying nanoparticle/plasmid DNA complexes encoding bFGF and VEGFA effectively transfected genes into tendon cells, significantly increasing growth factor expression in tendon tissues. Biomechanical testing showed that these gene-loaded sutures increased flexor tendon healing strength by 3.7-fold for non-absorbable sutures and 5.8-fold for absorbable sutures compared to unmodified controls. At 6 weeks, the nanoparticle/plasmid complex-coated sutures significantly increased tendon gliding excursions and inhibited adhesion formation [37]. Although these data derive from flexor tendon models rather than elbow-specific repairs, the suture technology is anatomically agnostic and is being extended toward tendon and ligament fixation constructs used about the elbow.

Established biologic treatments for lateral epicondylitis include platelet-rich plasma, autologous blood injections, and autologous tenocyte-cell injections derived from skin fibroblasts, the last of which produced significant improvement in Patient-Rated Tennis Elbow Evaluation scores at 6 weeks, 3 months, and 6 months in a pilot study of 12 patients with refractory disease, conducted by Hou et al. These cell-based approaches, while not nanotechnology per se, have created the biological rationale and clinical infrastructure onto which nanoscale delivery systems are now being layered [34,35,38].

Electrospun nanofiber scaffolds represent the most developed nanotechnology platform for elbow tendon regeneration. These scaffolds replicate the fibrillar architecture of native tendon ECM and allow precise control of fiber diameter, alignment, and porosity, properties that directly govern tenocyte orientation, proliferation, and matrix deposition [39]. Multifunctional nanofiber systems have been engineered to simultaneously modulate the inflammatory phase of healing, promote tenogenic differentiation of resident and implanted cells, enhance healing at the tendon-bone junction, and reduce the peritendinous scar and adhesion formation that commonly limits outcomes after epicondylar tendon debridement or repair [40]. Since these constructs are biodegradable and cell-instructive rather than purely structural, they are increasingly framed as a bridge between orthobiologic injections and formal surgical scaffold implantation for recalcitrant epicondylitis [39,40].

Nanophase gold has shown anti-inflammatory effects on tendon inflammation in preclinical models, suggesting a possible future adjunct for chronic tendinopathy refractory to first-line biologic and physical therapy management, though clinical data specific to the elbow are not yet available [1].

### 6.2. Targeted Drug Delivery for Tendon Healing

A sophisticated nanoparticle-based tendon-targeting drug delivery system has been developed to pharmacologically modulate tendon healing. This system leverages tartrate-resistant acid phosphatase (TRAP) activity, which is robustly expressed in healing tendons, to target TRAP-binding peptide (TBP)-functionalized nanoparticles specifically to injured tissue. When loaded with niclosamide (an S100a4 inhibitor), these targeted nanoparticles enhanced both functional and mechanical recovery in tendon injury models, while systemic delivery of free drug showed no benefit. This demonstrates the critical importance of targeted delivery for achieving therapeutic efficacy while minimizing systemic exposure [41].

Extracellular vesicle-cloaked enzymatic nanohybrids (ENEVs) represent another advanced approach for tendon regeneration. These multifaceted biocatalysts combine catalase-mimetic ROS scavenging, sustained zinc ion release, ultrasound-augmented cellular uptake, and immunoregulation induced by extracellular vesicles. In a rat Achilles tendon defect model, ENEVs combined with ultrasound treatment significantly promoted functional recovery and matrix reconstruction, restored tendon morphology, suppressed intratendinous scarring, and inhibited peritendinous adhesion. The ENEVs reversed macrophage polarization from M1 to M2 phenotype, creating a favorable immune microenvironment for healing [42].

### 6.3. Nanotechnology in Ulnar Collateral Ligament and Elbow Cartilage Regeneration

Beyond tendinopathy, orthobiologic strategies for the elbow and upper extremity encompass ulnar collateral ligament injury, osteochondral lesions (including osteochondritis dissecans of the capitellum), and osteonecrosis, with therapies spanning platelet-rich plasma, bone marrow aspirate concentrate and mesenchymal stromal cells, vascularized bone grafting, and engineered scaffolds combining osteoinductive beta-tricalcium phosphate and poly-epsilon-caprolactone with hydrogels and matrix metalloproteinases [1,3,5,9].

Bioactive nanostructured scaffolds engineered for tendon and ligament tissue engineering, particularly electrospun fibrous constructs incorporating growth factor delivery and cyclic mechanical stretching cues, are positioned as a future pathway for improving the historically inferior mechanical properties of repaired ligamentous tissue, including the ulnar collateral ligament, though current clinical evidence in this specific application remains at the level of expert opinion and preclinical modeling [2,4,6,10].

### 6.4. Total Elbow Arthroplasty and Fracture Fixation

While less extensively studied than shoulder applications, nanotechnology principles apply equally to elbow arthroplasty and fracture fixation. Total elbow arthroplasty shares the same nanoscale implant-engineering principles described for shoulder arthroplasty, but with added constraints related to the elbow’s smaller bone stock and higher inherent constraint forces. Nanotextured metal surfaces, produced by anodization, laser texturing, or severe plastic deformation of titanium, refine coarse metallic grains into the nanoscale range, improving both the biocompatibility and mechanical properties of the implant and enhancing osteoblast adhesion and osseointegration at the bone-implant interface. Polyethylene wear remains a specific concern in elbow arthroplasty because of the high constraint and contact stresses at the ulnohumeral articulation. Ultra-high molecular weight polyethylene (UHMWPE) has historically had limited use in constrained implants over fracture concerns, but the incorporation of carbon nanotubes into UHMWPE has produced a composite with improved mechanical strength and wear resistance while preserving favorable biocompatibility, with translational potential as a bearing surface in constrained or semi-constrained elbow prostheses. Broader nanostructured coating strategies applicable to elbow implants include nanostructured hydroxyapatite and diamond-like carbon coatings, which reduce abrasive wear while promoting osseointegration, and drug-eluting or metal-ion nanocoatings designed to combine antimicrobial and osteoinductive functions on a single implant surface [1,3,9].

Nanostructured titanium surfaces enhance osseointegration of elbow prostheses, potentially improving longevity and reducing aseptic loosening [2,4]. Antimicrobial nanocoatings may be particularly valuable in elbow arthroplasty given the relatively high infection rates (2–5%) compared to other joint replacements [25,26].

Nanostructured implant surfaces also apply to fracture fixation of the distal humerus, proximal radius and ulna, and olecranon, where anodization or laser texturing of plate and screw surfaces promotes more rapid and robust bone healing. Surface patterning at the nanoscale has been identified as an essential factor for biological response, achievable through anodization or laser texturing. Bioactive coatings incorporating growth factors, antimicrobial agents, or antiresorptive drugs can be applied to nanostructured surfaces to further enhance healing while preventing complications [33].

## 7. Mechanisms of Nanotechnology-Enhanced Healing

The success of nanotechnology in shoulder and elbow surgery derives from fundamental principles of cellular biology and biomaterial science. At the nanoscale, materials interact with biological systems in ways that are impossible to achieve with conventional microscales or macroscale structures [1,2].

The increased surface area-to-volume ratio of nanomaterials enables dramatically enhanced protein adsorption, which serves as the critical first step in cellular recognition and attachment [7,8]. Nanostructured surfaces selectively adsorb specific proteins (fibronectin, vitronectin, bone morphogenetic proteins) in conformations that optimize integrin binding and subsequent cellular signaling [8,22]. This selective protein adsorption creates a favorable microenvironment that directs stem cell differentiation toward desired lineages (osteogenic, tenogenic, chondrogenic) [6,43].

Nanotopography provides physical cues that guide cellular behavior through mechanotransduction pathways [5,43]. Aligned nanofibers direct cellular orientation and migration, promoting organized tissue regeneration rather than disorganized scar formation [5,6,44]. The diameter, spacing, and alignment of nanofibers can be precisely controlled to optimize specific cellular responses [43,45].

## 8. Where Are We Now?

### 8.1. Clinical Translation and Evidence Hierarchy

The translation of nanotechnology from bench to bedside in shoulder and elbow surgery has progressed through well-defined stages. Early preclinical studies in animal models established proof-of-concept and safety profiles [21,44,45]. Subsequent clinical studies, including prospective cohorts and RCTs, have demonstrated efficacy in human patients [9,10,13,18]. The current landscape of nanotechnology applications in upper extremity surgery, categorized by their biological mechanisms and clinical impacts, is summarized in Table 1.

**Figure 4:**
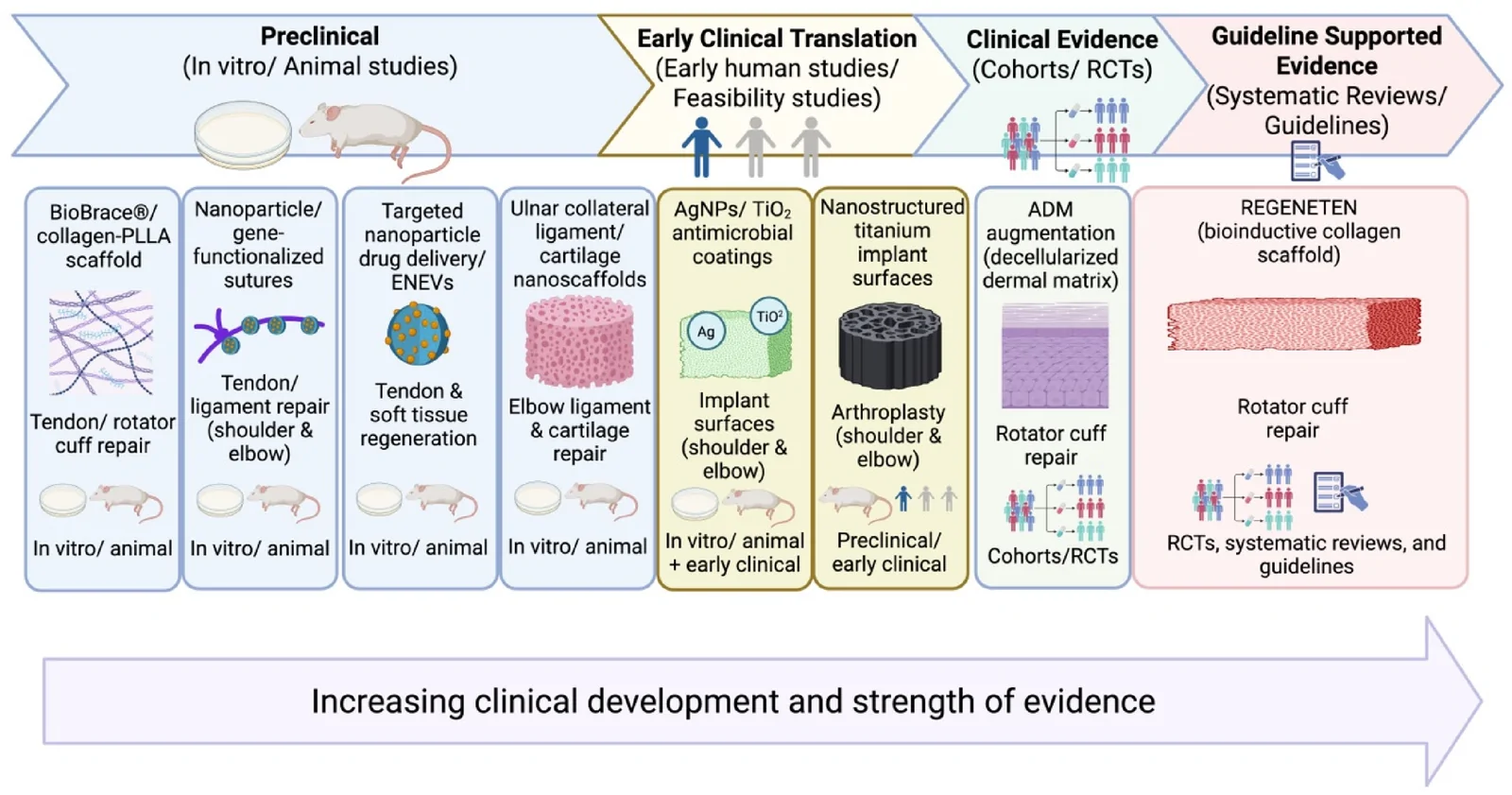
Translational landscape and levels of evidence for nanotechnologies in shoulder and elbow surgery. Technologies are organized along the translational continuum from preclinical evaluation (in vitro and animal models) to guideline-supported clinical evidence. ADM, acellular dermal matrix; AgNPs, silver nanoparticles; ENEVs, engineered extracellular vesicles; PLLA, poly-L-lactic acid; RCT, randomized controlled trial; TiO_2_, titanium dioxide. Created in BioRender. Pang, A.S.-R. (2026) https://BioRender.com/nxuik64 (accessed on 27 September 2026).

The evidence base is strongest for rotator cuff repair augmentation, where multiple RCTs and systematic reviews support the use of bioinductive collagen scaffolds and acellular dermal allografts, as illustrated in Figure 4 [9,10,11,14,16]. The AAOS 2025 guidelines provide moderate-strength recommendations for these technologies based on high-quality evidence [19].

For arthroplasty applications, the evidence remains primarily preclinical and early clinical, with long-term outcomes data emerging for specific implant systems [30,31]. The biological rationale is compelling, and in vitro and animal studies consistently demonstrate benefits, but large-scale clinical trials are needed to definitively establish superiority over conventional implants [7,23].

Across these elbow applications, the evidence hierarchy is consistent: nanostructured implant surface engineering for total elbow arthroplasty and fracture fixation rests on a substantial preclinical and cross-joint mechanistic literature but lacks elbow-specific clinical trials [1,3,5,7,9]. Elbow surgery applications represent the earliest stage of clinical translation, with most evidence derived from preclinical models and small clinical series [36,37,41,42]. The targeted drug delivery and gene therapy approaches show particular promise but require extensive additional study before routine clinical use [41]. Nanofiber and nanoparticle-functionalized suture technologies for tendon and ligament repair have supportive models in flexor tendon and general tendinopathy research but limited direct elbow clinical data [4,6,10]. Orthobiologic strategies for the ulnar collateral ligament and elbow cartilage remain at the level of expert opinion pending nanotechnology-specific clinical study [2]. Overall, elbow applications of nanotechnology are the least clinically mature of the domains reviewed, underscoring the need for elbow-specific prospective trials as these platforms move from bench to bedside.

The evidence synthesized in this review spans a heterogeneous range of study designs, and this heterogeneity carries important implications for how the reported outcomes should be interpreted. Retear rates, functional scores, and osseointegration metrics are drawn variably from RCTs, retrospective cohort studies, and preclinical animal or bench models, each with distinct risk-of-bias profiles that limit direct comparability. Surgical RCTs in this space are rarely blinded, given the inherent difficulty of masking the surgeon or, in some cases, the patient to the augmentation technique used, introducing potential performance and detection bias into subjective outcome measures such as pain and functional scoring. Preclinical and animal studies typically involve small sample sizes and short follow-up intervals, and biological or mechanical findings in these models do not necessarily translate proportionally to clinical outcomes in humans. Many of the device-specific studies, particularly those evaluating proprietary scaffolds and coatings, are industry-sponsored or involve authors with financial ties to the manufacturer, raising the possibility of publication or sponsorship bias.

Given these differences in study quality, sample size, and susceptibility to bias, the comparisons presented throughout this review, including retear risk reductions and preclinical mechanical benefits, should not be interpreted as equivalent-quality evidence or pooled as though derived from a single methodological standard. Rather, they should be read as directional signals from an evolving and methodologically heterogeneous evidence base, with the strength of any given claim weighted according to its underlying study design.

### 8.2. Safety Considerations and Regulatory Challenges

The safety profile of nanotechnology-based devices in shoulder and elbow surgery has been generally favorable, with low rates of adverse events directly attributable to nanomaterials [11,16,18]. However, several important considerations merit attention. Cytotoxicity concerns exist particularly for metallic nanoparticles such as silver [27,29]. While AgNPs demonstrate potent antimicrobial effects, they also generate reactive oxygen species that can damage eukaryotic cells [29]. Careful optimization of concentration, size, and release kinetics is essential to achieve antimicrobial efficacy while maintaining biocompatibility [27]. Gold nanoparticles may offer a better balance between antimicrobial activity and cytocompatibility due to their inability to generate ROS. Current cytotoxicity concerns can be anchored with specific numbers: AgNP-loaded hydroxyapatite/collagen composites show an IC50 for osteoblasts (MG-63) of 13.3 ± 0.02 µg/mL at 1 day and 7.85 ± 0.38 µg/mL at 7 days, roughly 7.5–13.8 times higher than the MICs against *E. coli* and *S. aureus* (0.96 ± 0.02 and 1.76 ± 0.06 µg/mL)—establishing a measurable, if narrowing, therapeutic window with prolonged exposure [46]. Conversely, other in vitro data show AgNPs (50 nm) are cytotoxic to osteoblasts and osteoclasts at concentrations only 2–4-fold above the antibacterial threshold for *S. epidermidis*, a narrower margin than the hydroxyapatite/collagen system [47]. Coating-based approaches report better separation: subnanomolar silver loading on titanium phosphonate monolayers preserved osteoblast attachment, proliferation, and differentiation while inhibiting *E. coli*/*S. epidermidis* biofilm in vivo, and silver/titanium nitride coatings at 7–18 at% controlled PJI pathogens while sustaining osteoblast metabolic activity to 28 days [48,49].

Long-term persistence and potential systemic distribution of nanoparticles remain incompletely characterized [29]. While most nanostructured surfaces involve fixed topographical features rather than free nanoparticles, wear debris from articulating surfaces could theoretically generate nano-sized particles [1]. The health effects of chronic exposure to such particles require ongoing surveillance [29].

Regulatory pathways for nanotechnology-based medical devices continue to evolve. In the United States, the Food and Drug Administration (FDA) evaluates these devices through the 510(k) or premarket approval (PMA) pathways depending on risk classification. Demonstrating substantial equivalence to predicate devices can be challenging when novel nanoscale features are involved. The European Union’s Medical Device Regulation (MDR) similarly requires comprehensive evaluation of safety and performance, with particular attention to novel materials and technologies [50].

### 8.3. Cost-Effectiveness and Healthcare Economics

The economic impact of nanotechnology in shoulder and elbow surgery represents an important consideration for healthcare systems and payers. While nanotechnology-enhanced devices typically cost more than conventional alternatives, the potential for reduced revision surgery rates may result in overall cost savings [16,51].

Two studies of bioinductive collagen implants for rotator cuff repair found cost savings of $5338–$13,061 per healed tendon due to reduced retear rates and associated revision procedures [16]. These analyses considered the upfront cost of the implant against the substantial costs of revision surgery, including the procedure itself, anesthesia, facility fees, rehabilitation, and lost productivity [16,51].

However, cost-effectiveness analyses remain limited, and concerns persist regarding the incremental cost relative to functional outcome improvements [51]. While structural healing (reduced retear rates) consistently improves with nanotechnology augmentation, functional outcomes (pain, range of motion, patient-reported outcome measures) do not always show proportional improvements [9,10,19]. This disconnect between structural and functional outcomes complicates value assessments and reimbursement decisions [51].

### 8.4. Future Directions and Emerging Technologies

The future of nanotechnology in shoulder and elbow surgery promises even more sophisticated applications. Several emerging areas warrant particular attention. First, smart scaffolds with controlled release. Next-generation scaffolds will incorporate multiple bioactive agents (growth factors, anti-inflammatory drugs, antimicrobials) with precisely controlled spatiotemporal release profiles [36,37,41]. Stimuli-responsive nanoparticles that release their cargo in response to specific triggers (pH, temperature, enzymes, ultrasound) will enable on-demand drug delivery [42].

Second, 3D bioprinting with nanomaterials. Additive manufacturing techniques combined with nanomaterial inks will enable creation of patient-specific scaffolds with hierarchical architecture spanning nano- to macro-scales [23]. These constructs can incorporate living cells, growth factors, and nanoparticles in precise spatial arrangements to guide tissue regeneration [4,32].

Third, nanorobots and targeted therapy. While still largely theoretical, nanorobots capable of navigating specific tissue sites and performing therapeutic functions represent a long-term vision for orthopedic nanotechnology [4,32]. However, their clinical translation faces substantial challenges, including reliable navigation and control within complex biological environments, biocompatibility and biodegradability, immune recognition and clearance, controlled therapeutic payload delivery, and the potential for unintended tissue accumulation or toxicity [52,53]. Additional barriers include manufacturing reproducibility, scalability, long-term safety assessment, and the absence of well-established regulatory pathways for autonomous or externally actuated nanoscale devices [54]. Consequently, nanorobotic applications in shoulder and elbow surgery should currently be regarded as conceptual or early preclinical technologies rather than near-term clinical interventions [52,53]. In contrast, targeted nanoparticle systems that deliver therapeutic agents to injured tissues through specific ligand-receptor interactions represent a more immediately translatable strategy [41].

Fourth, diagnostic applications in orthopedic surgery. Nanosensors capable of detecting biomarkers of inflammation, infection, or implant loosening could enable earlier intervention and improved outcomes [2,32]. Quantum dots and other fluorescent nanoparticles may enhance intraoperative imaging and surgical navigation [32].

Lastly, combination therapies integrating multiple nanotechnology approaches (nanostructured surfaces + antimicrobial coatings + growth factor delivery) will provide synergistic benefits addressing multiple aspects of healing simultaneously [22,24,33].

### 8.5. Challenges and Limitations of Nanotechnology

Despite the promise of nanotechnology in shoulder and elbow surgery, several challenges must be addressed to realize its full potential. First, standardization and reproducibility. Manufacturing processes for nanomaterials must achieve consistent quality and performance across production batches [50]. Variability in nanoparticle size, shape, surface chemistry, or scaffold architecture can significantly impact biological responses [7,23].

Second, the lack of long-term outcomes data. While short- and medium-term results are encouraging, long-term follow-up (i.e., 5–10 years) is needed to assess durability of nanotechnology-enhanced repairs and implants. Potential late complications such as chronic inflammation, particle-induced osteolysis, or carcinogenesis require ongoing surveillance [29].

Third, while empirical evidence supports nanotechnology applications, detailed mechanistic understanding of how specific nanoscale features influence cellular and tissue-level responses remains incomplete [6,7]. Better understanding will enable rational design of optimized materials rather than empirical trial-and-error approaches [8].

Fourth, the expense and complexity of clinical trial design remain prohibitive, particularly for small companies developing innovative technologies [50]. Demonstrating superiority of nanotechnology-enhanced devices requires large, well-designed RCTs with adequate follow-up duration.

Lastly, the lack of regulatory harmonization remains a pertinent challenge. Divergent regulatory requirements across different countries complicate global commercialization of nanotechnology-based devices. International harmonization of standards and approval pathways would facilitate broader access to beneficial technologies [50].

## 9. Conclusions

Nanotechnology has fundamentally transformed shoulder and elbow surgery through innovations spanning rotator cuff repair augmentation, arthroplasty osseointegration, antimicrobial coatings, and targeted drug delivery. The biomimetic approach of engineering materials at the nanoscale to replicate native tissue architecture has proven remarkably effective in enhancing healing and reducing complications. By engineering materials that replicate the nanoscale architecture of the native ECM, these innovations, such as bioinductive collagen scaffolds for rotator cuff repair and nanostructured titanium surfaces for arthroplasty, significantly enhance osseointegration, reduce repair failure, and promote a pro-healing immune microenvironment. While antimicrobial nanocoatings and targeted drug delivery systems offer promising solutions for infection control and complex tendinopathies, the field must still address challenges regarding manufacturing standardization, long-term safety data, and the current disconnect between improved structural healing and clinical functional outcomes. As regulatory pathways evolve, the integration of these nanoscale technologies remains essential for achieving superior, durable clinical results in upper extremity orthopedic care.

## Figures and Tables

**Table 1:** Summary of Nanotechnology Applications and Clinical Outcomes in Shoulder and Elbow Surgery.

Application Area	Specific Technology	Evidence Stage	Primary Mechanism of Action	Key Clinical/Preclinical Benefit(s)	Advantages	Limitations	References
Rotator Cuff	Bioinductive Collagen (REGENETEN)	Clinical (RCTs)	Induces new tissue via porous nanofibrous architecture	One RCT reported a 68% relative reduction in retear risk at 12 months (8.3% vs. 25.8%); subsequent studies support reduced retear rates, although effect estimates vary	Clinical evidence from RCTs; promotes biological augmentation without requiring structural graft replacement; improved tendon thickness and structural healing	Added implant cost; functional improvements may not parallel structural healing; limited long-term outcome data	[9,10,16,17,18]
Rotator Cuff	Biocomposite (BioBrace^®^)	Preclinical	Hybrid PLLA/collagen scaffold providing mechanical support and biological induction	180% increase in tendon thickness (preclinical)	Combines mechanical reinforcement with biologically supportive scaffold architecture; potentially useful in compromised tendon tissue	Evidence remains predominantly preclinical; human efficacy and long-term safety remain to be established	[20,21]
Shoulder Arthroplasty	Nanostructured Ti Surfaces	Preclinical	Enhanced surface area for protein adsorption/integrin binding	Accelerated osseointegration and vertical bone ingrowth	Promotes osteoblast adhesion and osseointegration; modifies the implant surface without requiring delivery of free nanoparticles	Limited shoulder-specific clinical evidence; long-term superiority over conventional implant surfaces remains uncertain	[1,7,8,22,23]
Shoulder Arthroplasty	Silver Nanoparticles (AgNPs)	Preclinical	ROS generation and bacterial membrane disruption	Broad-spectrum antimicrobial activity against MRSA/*E. coli*	Broad-spectrum antimicrobial activity; potential to reduce implant-associated bacterial colonization and biofilm formation	Dose-dependent cytotoxicity and narrow therapeutic window in some systems; concerns regarding particle persistence/systemic exposure; limited clinical validation	[25,27,28]
Elbow Surgery	Nanoparticle-coated Sutures	Preclinical	Sustained local delivery of bFGF and VEGFA	3.7- to 5.8-fold increase in healing strength in preclinical models	Localized and sustained therapeutic delivery at the repair site; may enhance biological healing while limiting systemic exposure	Evidence derived mainly from preclinical tendon models; limited direct elbow-specific evidence; manufacturing and delivery reproducibility require validation	[36,37]
Soft Tissue	Extracellular Vesicles (ENEVs)	Preclinical	ROS scavenging and M1-to-M2 macrophage polarization	Suppressed scarring and improved matrix reconstruction	Cell-free regenerative strategy; combines antioxidant and immunomodulatory effects; potential to promote a pro-healing microenvironment	Preclinical evidence only; challenges include standardization, characterization, manufacturing scalability, and reproducible delivery	[42]

## Data Availability

No new data were created or analyzed in this study. Data sharing is not applicable to this article.
